# A Unifying Hypothesis for Familial and Sporadic Alzheimer's Disease

**DOI:** 10.1155/2012/978742

**Published:** 2012-02-14

**Authors:** Carole J. Proctor, Douglas A. Gray

**Affiliations:** ^1^Centre for Integrated Systems Biology of Ageing and Nutrition, Institute for Ageing and Health, Newcastle University, Newcastle upon Tyne NE4 5PL, UK; ^2^Ottawa Hospital Research Institute, Ottawa, ON, Canada K1H 8L6; ^3^Department of Biochemistry, Microbiology and Immunology, University of Ottawa, Ottawa, ON, Canada K1H 8M5

## Abstract

Alzheimer's disease (AD) is characterised by the aggregation of two quite different proteins, namely, amyloid-beta (A**β**), which forms extracellular plaques, and tau, the main component of cytoplasmic neurofibrillary tangles. The amyloid hypothesis proposes that A**β** plaques precede tangle formation but there is still much controversy concerning the order of events and the linkage between A**β** and tau alterations is still unknown. Mathematical modelling has become an essential tool for generating and evaluating hypotheses involving complex systems. We have therefore used this approach to discover the most probable pathway linking A**β** and tau. The model supports a complex pathway linking A**β** and tau via GSK3**β**, p53, and oxidative stress. Importantly, the pathway contains a cycle with multiple points of entry. It is this property of the pathway which enables the model to be consistent with both the amyloid hypothesis for familial AD and a more complex pathway for sporadic forms.

## 1. Introduction

Alzheimer's disease (AD) is characterised by the presence of extracellular amyloid-beta (A*β*) plaques and cytoplasmic tau tangles and the loss of neurons in specific regions of the brain. The connection between these events is still not clear although it has been proposed that the formation of plaques precedes the appearance of tangles which in turn precedes cell death [1, 2]. Confounding the acceptance of such a simple temporal order of events is evidence that plaques are not necessary for disease progression [3] and that the accumulation of plaques can also occur as part of normal ageing with no apparent pathology [4]. Moreover, soluble A*β* may be a better correlate of disease than the insoluble plaques [5, 6]. It has recently been suggested that the amyloid hypothesis may only hold for familial forms of the disease but that the situation is much more complex in late-onset forms [7]. It is also possible that A*β* is a damage response protein [8]. Small and Duff [7]suggest that the pathway between A*β* and tau is linear for early-onset AD but hypothesize that a dual pathway links the two in late-onset disease [7]. A number of molecular pathways have been proposed as the upstream driver of both A*β* and tau aggregates. One important candidate is glycogen synthase kinase-3*β* (GSK3*β*). It is well established that GSK3*β* activity leads to hyperphosphorylation of tau and there is also evidence that it accounts for increased production of A*β* [9]. Its importance in AD was highlighted in 2008 by the proposal of a “GSK3 hypothesis of AD” [10]. A recent review also surveys data in support of the contention that GSK3*β* provides the link between A*β* and tau [11]. In addition it has been shown that A*β* behaves like an antagonist of insulin and prevents activation of Akt [12]. Akt phosphorylates GSK3*β* which inhibits its activity; A*β* therefore indirectly increases the activity of GSK3*β*. There is also a link between p53 and GSK3*β* and we recently modelled this to show that this interaction might explain the link between protein aggregation and neuronal loss in AD [13]. The model predicts that GSK3*β* overactivity leads to an increase in levels of A*β* plaques and tau tangles by independent processes supporting the idea of a dual pathway.

**Figure 1:**
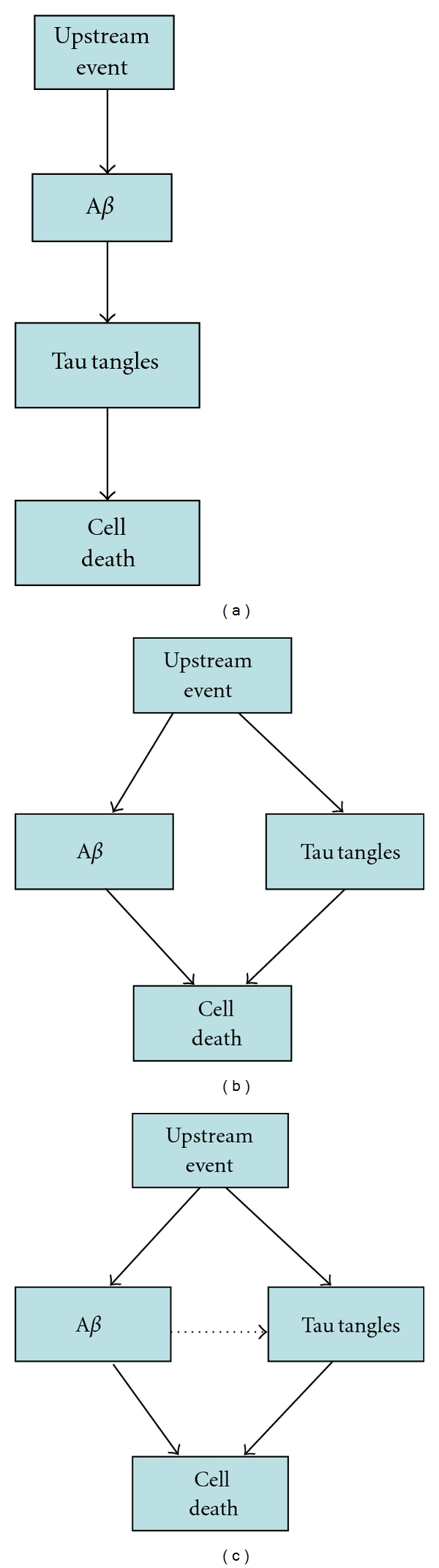
Alternative hypotheses for the link between A*β* and tau. (a) Linear pathway. (b) Dual pathway. (c) Complex pathway.

One way to examine the order of events in disease pathology is to prevent the formation of plaques and then observe whether or not tau tangles appear. An experimental procedure for doing this is A*β* immunization which has been carried out in many mouse models and also in a number of human clinical trials. Many of the mouse models do not have tau pathology and so cannot be used to test hypotheses concerning the order of events. In the more relevant 3×Tg-AD mouse model, experiments indicated that reducing plaques also led to the clearance of early tau pathology [14]. On the other hand human clinical trials have not shown any clear evidence of a reduction in tau tangles in regions where plaques were reduced [15]. Our model of GSK3/p53 [13] can examine the effect of increased clearance of A*β* by the simple modification of increasing the rate of A*β* (soluble form) removal. By doing so it is possible to test whether there is a linear or a dual pathway. If the pathway is linear, then the model should predict that increasing clearance of A*β* will also reduce the formation of tau tangles (Figure 1(a)). If there is a dual pathway, then increasing the clearance of A*β* will not affect the levels of tau tangles (Figure 1(b)). However, there is a third possibility (complex pathway): A*β* may not directly affect the formation of tau tangles in a linear pathway but may still have indirect effects (Figure 1(c)).

## 2. Methods

We previously built a stochastic dynamic model of p53 regulation [16] which was then extended to include GSK3*β*, A*β* and tau [13]. The models are encoded in the Systems Biology Markup Language (SBML), a computer-readable format for network models [17]. SBML allows models to be easily modified and extended and also enables sharing of models since the code is publicly available from the Biomodels database [18]. The extended GSK3 model includes a module for the DNA damage response which leads to elevated levels of p53, which can then bind to GSK3*β*. We assume that binding of GSK3*β* to p53 increased the activity of both proteins. The model includes a module for p53 turnover in which we assume that p53 binds to the E3 ligase Mdm2 and is then ubiquitinated and targeted for degradation by the 26S proteasome. Under normal (unstressed) conditions, both p53 and Mdm2 are kept at low basal levels. However, when cells are stressed and DNA damage occurs, p53 is phosphorylated and is then unable to bind to Mdm2 and so is no longer degraded. Therefore p53 levels increase. In addition, phosphorylation of p53 increases its activity. Full details of this module are already published [16]. Under normal conditions when p53 levels are low, it is unable to bind to GSK3*β* and so we assume that GSK3*β* activity is low when cells are not stressed.

The model also includes reactions for the production, clearance and aggregation of A*β*, and the phosphorylation/dephosphorylation and aggregation of tau. In addition we assume that A*β* results in increased generation of ROS and increased transcription of p53. The full details of the model are available in an open access journal and the SBML code is available from Biomodels (BioModels ID:BIOMD0000000286)[18]. The simulations were carried out using the Gillespie algorithm on the Biology of Ageing e-Science Integration and Simulation (BASIS) system [19–21]. The model results were analysed and plotted using the R package.

## 3. Results

### 3.1. Increased A*β* Clearance from Day 0

**Figure 2:**
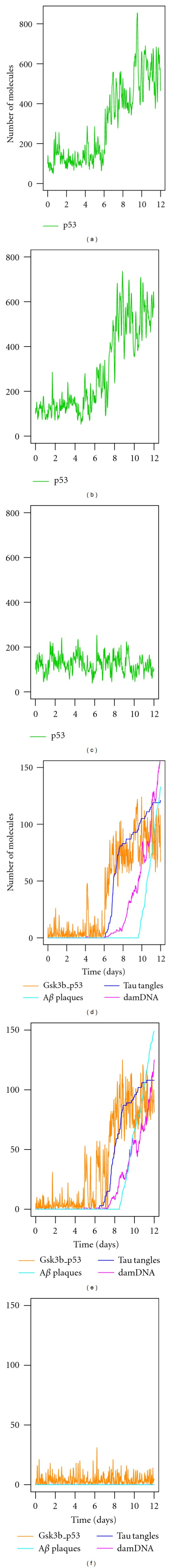
Simulation output for model with high aggregation rates and normal A*β* clearance. Three different individual simulations are shown. The plots in each column are from the same simulation. (a)–(c) Levels of p53 (total pool including bound and ubiquitinated species). (e,f) p53 bound to GSK3*β* (GSK3b_p53), damaged DNA (damDNA), tau tangles, and A*β* plaques are shown.

**Figure 3:**
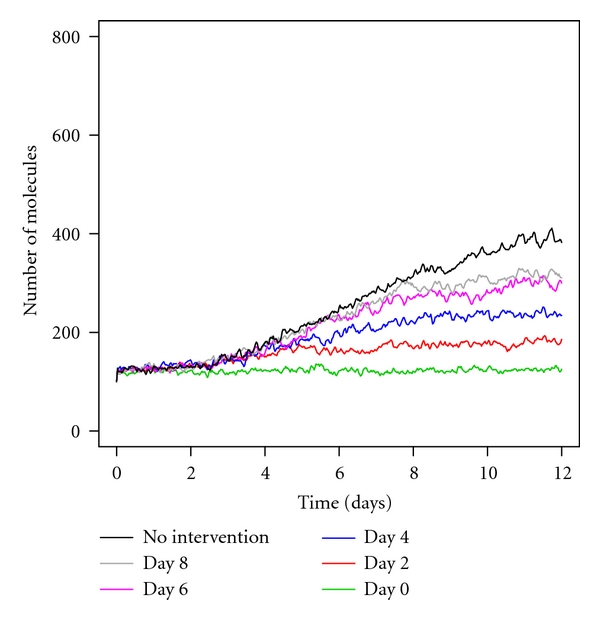
Levels of p53 under conditions of high aggregation rates and increased A*β* clearance at different time points. Each line shows the mean levels of p53 (total pool including bound and ubiquitinated species) from 100 simulations over a 12-day period.

**Figure 4:**
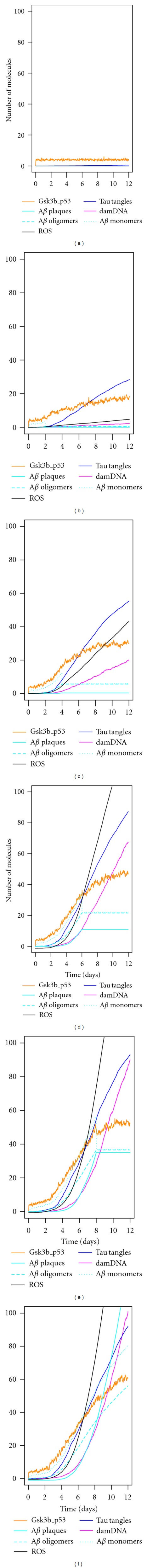
Simulation results for model with high aggregation rates and increased A*β* clearance at different time points. Each graph shows the mean of 100 simulations. The clearance rate of A*β* was increased at the following time points: (a) Day 0, (b) Day 2, (c) Day 4, (d) Day 6, (e) Day 8, (f) No intervention. p53 bound to GSK3*β* (GSK3b_p53), damaged DNA (damDNA), ROS, tau tangles and A*β* monomers, oligomers and plaques, are shown.

In our previous model we set the rates for aggregation of tau and A*β* at levels so that if there was an increase in tau phosphorylation or an increase in A*β* production, the formation of aggregates would appear within 2 or 3 days. In reality, the aggregation process is likely to have much longer lag periods. Acceleration of the aggregation process in our computer model is merely a device to increase the throughput of simulations. With normal rates of A*β* clearance, our model predicts that a small percentage of cells do not accumulate any plaques or tangles by 12 days (Figures 2(c) and 2(f)). However, the majority of simulated cells accumulate both plaques and tangles due to stochastic DNA damage which leads to increased levels and activation of p53 (Figures 2(a), 2(b), 2(d) and 2(e)). The model predicts that as a result of p53 activation, GSK3*β* activity increases resulting in increased phosphorylation of tau and formation of tau tangles. In addition, increased p53 and GSK3*β* activity result in increased production of A*β* which then aggregates to form plaques. Interestingly, the model predicts that tau tangles precede A*β* plaques suggesting that plaques and tangles are formed independently. The increase in A*β* also leads to more ROS and further DNA damage which in turn leads to further activation of p53 and a cycle ensues. Increasing the clearance rate of A*β*, by two orders of magnitude, at day 0 prevents any accumulation of plaques or tangles and p53 levels remain low over a simulated 12-day period (Figure 3, green curve and Figure 4(a)). This supports the hypothesis that the increase in ROS via A*β* reinforces the cycle by activation of p53 and GSK3*β* as suggested above.

### 3.2. Effect of Increasing A*β* Clearance at Different Time Points

It is of interest to examine the effect of increasing A*β* clearance at later timepoints, since such interventions may occur after soluble A*β* or even plaques have had time to form. Studies on A*β* immunization in mice indicate that interventions are more effective if administered early, suggesting that the load of A*β* at the time of immunization is important [22]. We therefore used the model to explore the effect of increasing the clearance of A*β* at different time points (Figures 3 and 4). This was done by adding a timed event to the SBML code so that the parameter for A*β* clearance is reduced by two orders of magnitude at time 2, 4, 6, or 8 days from the start of the simulation. The model predicts that increasing A*β* clearance at early time points (up to day 4) leads to a much lower level of A*β* so that no plaques form and there are also much lower levels of tau tangles and p53 (Figures 3, 4(a)–4(c)). Note that the intervention at day 2 leads to low levels of A*β* monomers which are sufficient to slightly increase ROS levels (black curve in Figure 4(b)). Accordingly p53 levels rise slightly (red line in Figure 3) and the activity of GSK3*β* is increased leading to an increase in phosphorylation and aggregation of tau (Figure 4(b)). Interventions at later time points (day 6 or later) result in lower levels of plaques compared to normal A*β* clearance (compare light blue curves in Figures 4(d) and 4(e) with 4(f)) but the levels of tau tangles are not significantly lower compared to no intervention (Figures 4(d)–4(f), dark blue curves). This is due to the formation of A*β* monomers and oligomers occurring before the intervention, which leads to increases in ROS, activation of GSK3*β*, and increased phosphorylation of tau which is then more likely to form tangles. Figure 3 shows p53 levels start to increase after day 2 and continue to increase until the intervention of increased A*β* clearance occurs. This can be seen clearly by the fact that all curves are initially close together but as the intervention occurs, p53 levels stabilise. The model therefore suggests that even a low level of soluble A*β* monomers and oligomers is sufficient to trigger an increase in ROS, which leads to an increase in p53.

### 3.3. Inhibition of ROS Production via A*β*

**Figure 5:**
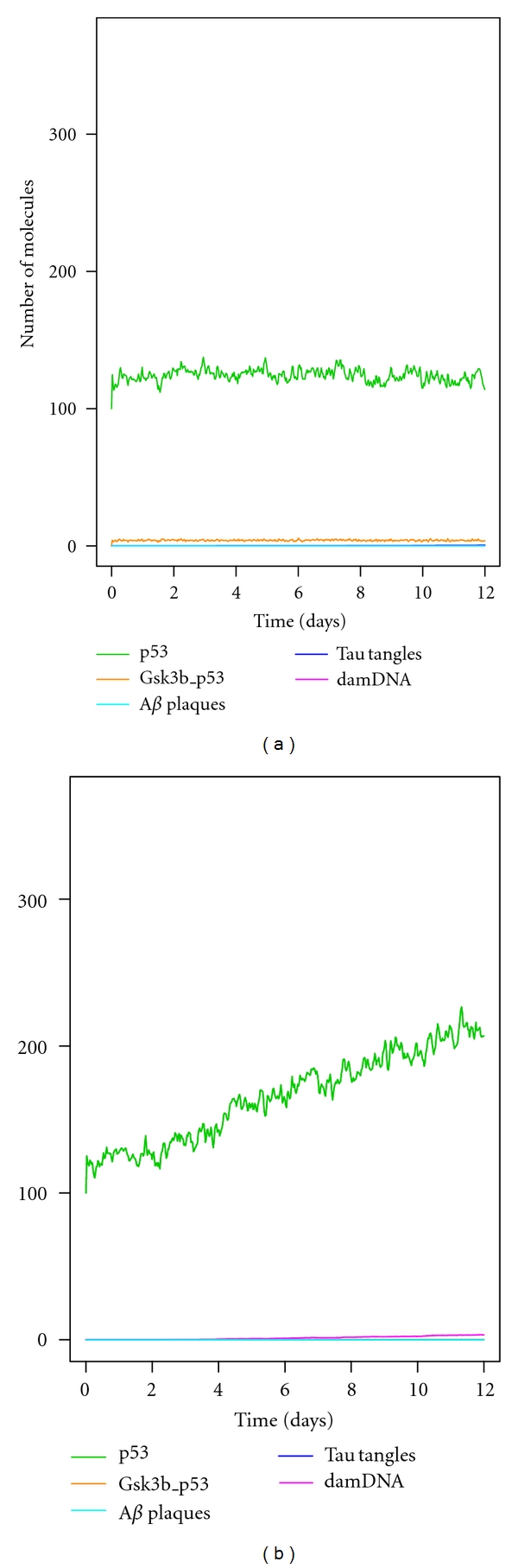
Increased A*β* clearance on day 8 with additional interventions. Each graph shows the mean of 100 simulations. (a) Blockage of ROS production via A*β* (parameter for A*β*-mediated ROS production set to zero). (b) Inhibition of GSK3*β*/p53 binding (parameter for GSK3*β*/p53 binding set to zero). Note that apart from p53, all proteins shown in the graphs have levels close to zero and so not all the lines can be seen.

To confirm whether the increase in p53 is due to A*β*-mediated ROS production, we ran 100 simulations in the model with increased A*β* clearance at day 8 and blocked the production of ROS via A*β* (by setting the parameter for A*β*-mediated ROS production to zero).  Figure 5(a) shows the mean value of these simulations for p53, GSK3*β* bound to p53, A*β* plaques, tau tangles, and damaged DNA over a 12-day period. It can be seen that with the exception of p53, the levels of the all species shown are close to zero. So the model predicts that this intervention completely prevents the increase in DNA damage, the elevation of p53, the increase in GSK3*β* activity, and the formation of plaques and tangles producing results similar to increased clearance of A*β* at day 0 (see Figure 4(a)).

### 3.4. Inhibition of GSK3*β*/p53 Binding

To examine the effect of GSK3*β*/p53 binding on the aggregation process we inhibited the interaction between GSK3*β* and p53 (by setting the parameter for GSK3*β*/p53 binding to zero). We ran 100 simulations with increased clearance of A*β* on day 8 (with ROS production via A*β* restored). This additional intervention also prevented the formation of plaques and tangles even though p53 levels rose during the simulation (Figure 5(b)). Therefore the model predicts that A*β* clearance at late time points may be beneficial if additional interventions are used such as simultaneously reducing ROS levels or preventing the activation of GSK3*β*.

### 3.5. Effect of A*β* Immunization on Neuronal Loss

**Figure 6:**
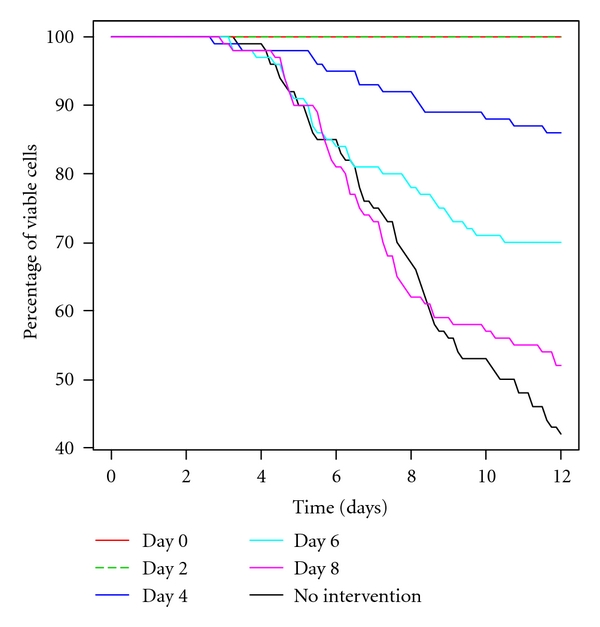
Percentage of viable simulated cells for increased A*β* clearance at different time points. Each curve shows how the percentage of viable cells (from 100 simulations) changes with time over a 12-day period when A*β* clearance is increased at days 0, 2, 4, 6, or 8 and for the normal clearance rate (no intervention).

Cell death is not currently explicitly included in the model, but we can assume that if p53 reaches a threshold then it triggers an apoptotic pathway. Since it would be unrealistic to assign to the threshold an exact and invariable value, the threshold level of p53 is chosen from a random distribution (normal distribution, mean 600, variance 50) for each simulation run. For each simulation the level of p53 was tracked over time, starting at time zero. If the level of p53 exceeded the chosen threshold, the time at which this occurred was recorded and the simulated cell was considered to have undergone cell death at this time. The percentage of viable cells at each time point was calculated for each of the intervention times and plotted (Figure 6). The model predicts that there are no cell deaths if A*β* clearance is increased at early time points but as the intervention is increasingly delayed the percentage of cell death increases. If the intervention is as late as day 8, there is little improvement in cell viability compared to no intervention. The model therefore indicates that increased clearance of A*β* needs to occur at early time points before there is any accumulation of A*β*.

## 4. Discussion

**Figure 7:**
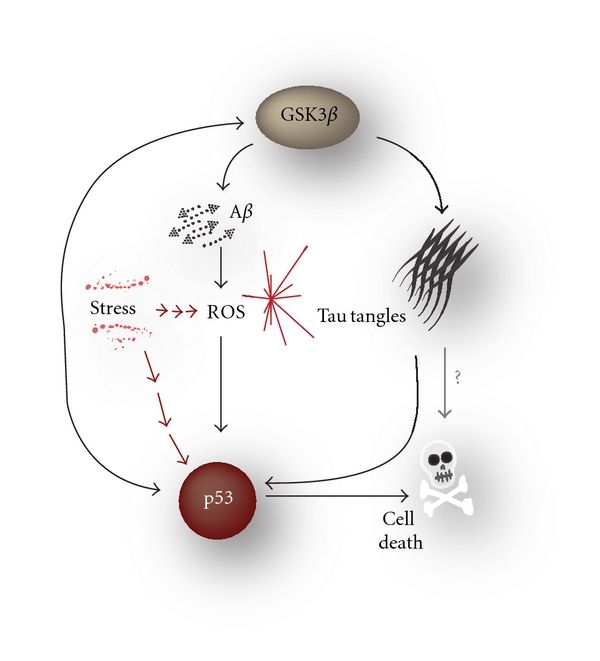
New hypotheses of AD involving GSK3 and p53. Our model supports that the pathway between A*β* and tau is via ROS, p53, and GSK3*β*. Note that there is a cycle in the diagram between GSK3*β*, A*β*, ROS, and p53 which can start at any point. Full details are in the text.

The model shows that reducing the burden of A*β* reduces levels of ROS, which leads to less DNA damage, lower p53 activity, lower GSK3*β* activity, and reduced tau phosphorylation. If A*β* clearance is increased at early time points, there is a decrease in plaques and also a reduction in tau tangles. The model therefore does not support a dual pathway (Figure 1(b)). On the other hand, increasing A*β* clearance at late time points reduced plaque formation but did not reduce tangle formation. Neither then does the model support a linear pathway (Figure 1(a)). Rather the model supports the complex pathway where plaques and tangles can form independently due to an upstream event but with increased tangle formation in the presence of A*β* (Figure 1(c)). We propose a new hypothesis in which the pathway between A*β* and tau is via ROS, p53, and GSK3*β* (Figure 7). It is important to note that GSK3*β*, which is shown at the top of the diagram, is not necessarily the starting point for the ensuing cascade of events. For example, the initiating event could be an increase in soluble A*β* which then leads to plaques and an increase in ROS. Elevated ROS may then cause DNA damage which results in increased levels of p53, followed by increased activity of GSK3*β*. Finally the increased activity of GSK3*β* leads to tau hyperphosphorylation and tangle formation. In addition, levels of A*β* are increased and so there is a positive feedback loop which reinforces the cycle on the left. Note that GSK3*β* also increases p53 activity providing an additional positive feedback in the cycle. The cycle could also begin with increased ROS due to cellular stress, an increase in dysfunctional mitochondria, and/or a decline in the efficiency of the antioxidant system. Furthermore, the cycle could begin with p53 due to stress-induced DNA damage, telomere uncapping, or inhibition of the proteasome. Whatever the initiating event the positive feedback loops could promote a self-perpetuating and amplifying cascade of events that could lead to frank AD.

The model also supports the amyloid hypothesis for familial forms of the disease, since the initiating event for this form of the disease would be increased production of A*β* due to mutations in genes involved in APP processing. In this case the cycle starts with A*β* and then leads to increased ROS, DNA damage, increased levels of p53, increased GSK3*β* activity, and finally hyperphosphorylation of tau and formation of tangles in a seemingly linear pathway. The model also explains why tau pathology may be seen before plaques or even without plaques if the initiating event is increased activity of GSK3*β*, or if the cycle starts with ROS or p53. The scenario in which tangles appear without any plaques would suggest however that there must also be more efficient clearance of A*β* since an increase in GSK3*β* activity also increases A*β* production.

There is experimental data to support all the arrows in the diagram, however the importance of p53 in the loop has not been fully investigated. Although it is known that p53 increases the activity of GSK3*β* [23] and that increased p53 activity indirectly leads to tau hyperphosphorylation [24], as yet no experiments have been carried out to prove that the link between p53 and tau is GSK3*β* as our model suggests. This prediction could be tested experimentally by either inhibiting or overexpressing p53 in cells expressing A*β* and then measuring GSK3*β* activity and levels of phospho-tau.

The model is a simplification of the system but as the model is encoded in SBML, it can easily be extended to include further details. Other important components which could be added are chaperones (GSK3*β* is a client of Hsp90), more detail of tau regulation, the insulin pathway, and wnt signalling pathways. It would be of particular interest to include the insulin signalling pathway in order to explore the connection between AD and type 2 diabetes since GSK3*β* has been implicated in both diseases. Mitochondria also play an important role in the disease process. For example, damaged mitochondria may accumulate in postmitotic neurons and cause an increase in ROS which could start the vicious cycle shown in Figure 7. In addition, soluble A*β* binds to A*β*-binding alcohol dehydrogenase (ABAD) which leads to increase ROS via mitochondrial dysfunction [25, 26]. Recent data show that truncated tau and A*β* act cooperatively to impair mitochondrial function and reduce mitochondrial transport in neurons [27]. A model of mitochondrial dynamics is currently being developed and linking this with the current model will give a more complete picture of the disease process.

A*β* immunotherapy works by either active immunization with A*β* aggregates or by passive transfer of anti-A*β* antibodies. Both approaches have been shown to prevent A*β* deposition and to clear already existing plaques. Wilcock et al. showed two phases in the clearance of plaques [28]. First, there was a sharp decline in plaques 24 hours after immunization due to disaggregation and then a further decline about 3 days later due to the activation of microglia which removed the plaques by phagocytosis [28]. Our current model could be modified to mimic the immunization process by including additional reactions for plaque disaggregation and clearance. The disaggregation of plaques leads to an increase in soluble A*β* and since these may be toxic due to their interaction with mitochondria and their involvement in ROS production, our model may show that such an intervention would be less beneficial than the increased clearance of soluble A*β*. Therefore, the model could prove very useful for testing the consequences of different interventions.

## 5. Conclusions

Our mathematical model supports a complex pathway linking A*β* and tau via GSK3*β*, p53, and oxidative stress. Importantly, the pathway contains a cycle with multiple points of entry. It is this property of the pathway which enables the model to be consistent with both the amyloid hypothesis for familial AD and a more complex pathway for sporadic forms.

## References

[1] Hardy JA, Higgins GA (1992). Alzheimer’s disease: the amyloid cascade hypothesis. *Science*.

[2] Hardy J, Allsop D (1991). Amyloid deposition as the central event in the aetiology of Alzheimer’s disease. *Trends in Pharmacological Sciences*.

[3] Terry RD, Masliah E, Salmon DP (1991). Physical basis of cognitive alterations in Alzheimer’s disease: synapse loss is the major correlate of cognitive impairment. *Annals of Neurology*.

[4] Katzman R, Terry R, DeTeresa R (1988). Clinical, pathological, and neurochemical changes in dementia: a subgroup with preserved mental status and numerous neocortical plaques. *Annals of Neurology*.

[5] Lue LF, Kuo YM, Roher AE (1999). Soluble amyloid *β* peptide concentration as a predictor of synaptic change in Alzheimer’s disease. *American Journal of Pathology*.

[6] McLean CA, Cherny RA, Fraser FW (1999). Soluble pool of A*β* amyloid as a determinant of severity of neurodegeneration in Alzheimer’s disease. *Annals of Neurology*.

[7] Small SA, Duff K (2008). Linking A*β* and tau in late-onset Alzheimer’s disease: a dual pathway hypothesis. *Neuron*.

[8] Hardy J (2009). The amyloid hypothesis for Alzheimer’s disease: a critical reappraisal. *Journal of Neurochemistry*.

[9] Sofola O, Kerr F, Rogers I (2010). Inhibition of GSK-3 ameliorates A*β* pathology in an adult-onset Drosophila model of Alzheimer’s disease. *PLoS Genetics*.

[10] Hooper C, Killick R, Lovestone S (2008). The GSK3 hypothesis of Alzheimer’s disease. *Journal of Neurochemistry*.

[11] Hernández F, de Barreda EG, Fuster-Matanzo A, Lucas JJ, Avila J (2010). GSK3: a possible link between beta amyloid peptide and tau protein. *Experimental Neurology*.

[12] Lee HK, Kumar P, Fu Q, Rosen KM, Querfurth HW (2009). The insulin/Akt signaling pathway is targeted by intracellular *β*-amyloid. *Molecular Biology of the Cell*.

[13] Proctor CJ, Gray DA (2010). GSK3 and p53—is there a link in Alzheimer’s disease?. *Molecular Neurodegeneration*.

[14] Oddo S, Billings L, Kesslak JP, Cribbs DH, LaFerla FM (2004). A*β* immunotherapy leads to clearance of early, but not late, hyperphosphorylated tau aggregates via the proteasome. *Neuron*.

[15] Nicolll JAR, Wilkinson D, Holmes C, Steart P, Markham H, Weller RO (2003). Neuropathology of human Alzheimer disease after immunization with amyloid-*β* peptide: a case report. *Nature Medicine*.

[16] Proctor CJ, Gray DA (2008). Explaining oscillations and variability in the p53-Mdm2 system. *BMC Systems Biology*.

[17] Hucka M, Finney A, Sauro HM (2003). The systems biology markup language (SBML): a medium for representation and exchange of biochemical network models. *Bioinformatics*.

[18] le Novère N, Bornstein B, Broicher A (2006). BioModels database: a free, centralized database of curated, published, quantitative kinetic models of biochemical and cellular systems. *Nucleic Acids Research*.

[19] Duke DC, Moran LB, Kalaitzakis ME (2006). Transcriptome analysis reveals link between proteasomal and mitochondrial pathways in Parkinson’s disease. *Neurogenetics*.

[20] Gillespie DT (1977). Exact stochastic simulation of coupled chemical reactions. *Journal of Physical Chemistry*.

[21] Kirkwood TBL, Boys RJ, Gillespie CS, Proctor CJ, Shanley DP, Wilkinson DJ (2003). Towards an e-biology of ageing: integrating theory and data. *Nature Reviews Molecular Cell Biology*.

[22] Schenk D, Barbour R, Dunn W (1999). Immunization with amyloid-*β* attenuates Alzheimer disease-like pathology in the PDAPP mouse. *Nature*.

[23] Watcharasit P, Bijur GN, Song L, Zhu J, Chen X, Jope RS (2003). Glycogen synthase kinase-3beta (GSK3beta) binds to and promotes the actions of p53. *Journal of Biological Chemistry*.

[24] Hooper C, Meimaridou E, Tavassoli M, Melino G, Lovestone S, Killick R (2007). p53 is upregulated in Alzheimer’s disease and induces tau phosphorylation in HEK293a cells. *Neuroscience Letters*.

[25] Lustbader JW, Cirilli M, Lin C (2004). ABAD directly links A*β* to mitochondrial toxicity in Alzheimer’s disease. *Science*.

[26] Takuma K, Yao J, Huang J (2005). ABAD enhances A*β*-induced cell stress via mitochondrial dysfunction. *FASEB Journal*.

[27] Quintanilla RA, Dolan PJ, Jin YN, Johnson GVW (2012). Truncated tau and A*β* cooperatively impair mitochondria in primary neurons. *Neurobiology of Aging*.

[28] Wilcock DM, DiCarlo G, Henderson D (2003). Intracranially administered anti-A*β* antibodies reduce *β*-amyloid deposition by mechanisms both independent of and associated with microglial activation. *Journal of Neuroscience*.

